# Impact of Subclinical Hypothyroidism on Lipid Profile in Jeddah: A Retrospective Cohort Study

**DOI:** 10.7759/cureus.65433

**Published:** 2024-07-26

**Authors:** Mahmoud A Alzahrani, Fatemah S Baqar, Basil A Alzahrani, Ziyad A Badri, Rayan Alshamrani, Jamal Aljuhani

**Affiliations:** 1 College of Medicine, King Saud Bin Abdulaziz University for Health Sciences, Jeddah, SAU; 2 King Saud Bin Abdulaziz University for Health Sciences, College of Medicine, King Abdullah International Medical Research Center, Jeddah, SAU

**Keywords:** subclinical hypothyroidism, low-density lipoprotein (ldl), hypothyroidism, subclinical, lipids, thyroid

## Abstract

Background

Patients with subclinical hypothyroidism (SCH) have a high serum concentration of thyroid-stimulating hormone (TSH), whereas their serum-free thyroxine concentrations are normal. Lipid metabolism is regulated in large part by thyroid hormones. It could be connected to a changed lipid profile. This study aimed to evaluate the relationship between SCH and alterations in the lipid profile.

Methodology

Data from 99 patients with SCH and 109 euthyroid cases were collected from King Abdulaziz Medical City, Jeddah, Saudi Arabia, from 2016 to 2022. Patients older than 18 years were included in the study. The groups were matched in terms of gender, age, and body mass index. SCH was defined as a TSH value of 4.5 to 10 mIU/L, and normal T4 as 5 to 18 μg/dL. Control cases had a normal TSH ranging from 0.45 to 4.5 mIU/L. The total serum cholesterol, high-density lipoprotein (HDL) cholesterol, low-density lipoprotein (LDL) cholesterol, and triglyceride (TG) levels in both groups were examined and the results were recorded.

Results

In comparison to the control group, SCH patients had greater median glycated hemoglobin (HbA1C) (p = 0.001) and lower median vitamin D levels (p = 0.004) before therapy. Before therapy, SCH patients also showed considerably lower HDL levels and significantly higher LDL and TG levels (p < 0.001).

Conclusions

There is a substantial correlation between SCH and reduced HDL and vitamin D levels. It was linked to increased TG, LDL, and HbA1c levels. Only vitamin D and LDL were pathologically high. Treatment with levothyroxine raised total and LDL cholesterol levels. Future research should look into the affordability of treating SCH.

## Introduction

Subclinical hypothyroidism (SCH) is characterized by the absence of distinct clinical symptoms and signs. Thyroid-stimulating hormone (TSH) levels in the serum of SCH patients were found to be high whereas serum-free thyroxine concentrations were normal. TSH levels can be used to categorize SCH into mild and severe forms. Mild SCH is defined as a TSH level less than 10 mIU/L (normal thyroxine), whereas severe SCH is defined as a TSH level greater than or equal to 10 mIU/L [1]. In the general population, the prevalence of SCH ranges from 4% to 8%, and in women over 60, it can reach 15% to 18% [2]. In a study conducted in outpatient clinics at a Jeddah university hospital, 35% of 257 Saudi women who participated had SCH [3]. Patients with SCH are typically only treated if they display related symptoms, are infertile, are pregnant, or have a high risk of developing overt hypothyroidism. SCH has a high prevalence, but the advantages and hazards of its therapy, as well as the data supporting screening for this disorder, are still up for debate [4].

Both primary and secondary hypothyroidism are frequently associated with hyperlipidemia. After receiving therapy for hypothyroidism, the lipid levels decrease. Patients with primary hypothyroidism are more likely to have type IIa hyperlipidemia than secondary hypothyroidism patients, who are more likely to have type IIb hyperlipidemia [5]. Moreover, according to a meta-analysis, patients with SCH had significantly higher serum levels of total cholesterol (TC), low-density lipoprotein cholesterol (LDL-C), and total triglycerides (TGs) than people with euthyroidism, with no significant difference in serum high-density lipoprotein cholesterol (HDL-C) [6]. Another systematic analysis demonstrated the significance of treating individuals with SCH by finding that levothyroxine therapy significantly reduced serum levels of TSH, TC, and LDL-C [7].

Recent clinical research has found a strong link between TSH and lipid metabolism as well as several cardiovascular illnesses. The effect of TSH on blood lipids has always been attributed in these investigations to thyroid hormone levels. Furthermore, findings from experimental trials provide substantial support for the concept that TSH directly affects lipid constituents [8]. Important physiological processes, including development, growth, and metabolism, are mediated by the thyroid hormone. The active form of TH is intracellular triiodothyronine (T3), which binds to the thyroid hormone receptor (TR), a transcription factor that is a member of the nuclear receptor superfamily. TRa and TRb are the two isoforms of the TR. In contrast to TRb, which predominates in the liver, TRa is highly expressed in the heart, muscle, and adipose tissue. Significant changes in body weight, thermogenesis, and lipolysis are caused by TH. These changes are predominantly mediated by the effects of thyroid hormone on skeletal muscle and adipose tissue. Through its effects on the liver, thyroid hormones can also control fatty acid, cholesterol, and glucose balance. Thus, hepatic lipid and carbohydrate metabolism can be negatively impacted by thyroid dysfunctions, which can also lead to intrahepatic and systemic dysregulation of the metabolism of substances that are critical sources of energy for cells [9]. Thyroid hormone enhances the flow of bile acids, depleting intrahepatic cholesterol while stimulating liver cholesterol synthesis and hepatic uptake of circulating cholesterol, maintaining the balance of hepatic cholesterol [10]. This study aimed to evaluate the impact of SCH on the lipid profile in adults.

This article was previously posted to the Research Square preprint server on November 30, 2023.

## Materials and methods

This case-control, retrospective, cohort study was conducted among patients followed in King Abdulaziz Medical City, Jeddah, Saudi Arabia, from 2016 to 2022. The study was approved by the Institutional Review Board of King Abdullah International Medical Research Center (approval number: NRJ23J/228/09). The main objective was to evaluate the impact of SCH on the lipid profile in adult patients. The secondary objective was to compare the lipid profile after treatment. The study included 208 patients divided into 99 patients in the affected group and 109 in the control group. The inclusion criteria were patients over 18 years old who were diagnosed with subclinical hypothyroidism. The exclusion criteria included patients who had a history of dyslipidemia, pregnancy, cancer, liver diseases, end-stage kidney disease, and diabetes.

Definitions

SCH was defined as TSH higher than 4.5 mIU/L, with normal T4 level defined as 5-18 μg/dL, total cholesterol as >5.18 mmol/L, LDL as >1.55 mmol/L, HDL as <1.55 mmol/L, and TG as >1.77 mmol/L.

Statistical analysis

Data were collected in Excel (Microsoft Corp., Redmond, WA, USA) and analyzed using R software (version 4.2.2). Normality was tested with histograms. Continuous variables were represented by mean and standard deviation and categorical variables by frequencies and percentages. The Wilcoxon rank sum test was used to compare clinical characteristics and lipid profiles between the SCH and control groups. Additionally, within the SCH group, Wilcoxon signed-rank tests were used to assess lipid profile differences before and after treatment. Statistical significance was set at p-values <0.05.

## Results

This study involved 208 patients, with a median age of 45 years. The study participants were predominantly male (51%) (N = 107) and obese (41%) (N = 101). Common comorbidities included hypertension. Patients with SCH were older and more obese than the control group (Table 1). Before treatment, SCH patients had lower median vitamin D levels (p = 0.004) and higher median glycated hemoglobin (HbA1C) (p = 0.001) compared to the control group. Additionally, SCH patients exhibited significantly higher LDL and TG levels, as well as significantly lower HDL levels before treatment (p < 0.001) (Table 2).

**Table 1:** Demographic characteristics of the participants. Median (IQR); n (%). BMI = body mass index; IQR = interquartile range

Characteristic	Overall, N = 208	Affected group, N = 99	Controlled group, N = 109
Age (years)	45 (35, 60)	50 (35, 67)	43 (36, 53)
Gender			
Female	101 (49%)	49 (49%)	52 (48%)
Male	107 (51%)	50 (51%)	57 (52%)
Comorbidities			
Hypertension	10 (4.8%)	4 (4.0%)	6 (5.5%)
Ischemic heart disease	8 (3.8%)	6 (6.1%)	2 (1.8%)
Others	14 (6.7%)	7 (7.1%)	7 (6.4%)
Missing	164		
BMI (kg/m^2^)			
Underweight	11 (5.3%)	4 (4.0%)	7 (6.4%)
Healthy weight	57 (27%)	19 (19%)	38 (35%)
Overweight	54 (26%)	26 (26%)	28 (26%)
Obese	86 (41%)	50 (51%)	36 (33%)

**Table 2:** Clinical characteristics of the participants among case and control groups. Median (IQR), Wilcoxon rank sum test. HbA1C = glycated hemoglobin; IQR = interquartile range

Characteristic	N	Overall, N = 208	Affected group, N = 99	Controlled group, N = 109	P-value
Vitamin D-25 before treatment	117	56 (36, 77)	44 (30, 70)	60 (40, 86)	0.004
Fasting glucose before treatment	145	5.10 (4.80, 5.60)	5.10 (4.80, 5.80)	5.05 (4.80, 5.40)	0.10
HbA1C	183	5.20 (5.00, 5.50)	5.40 (5.10, 5.90)	5.20 (5.00, 5.30)	0.001
Systolic blood pressure	178	128 (117, 138)	130 (115, 142)	124 (118, 137)	0.3
Diastolic blood pressure	178	74 (67, 80)	73 (69, 80)	75 (67, 80)	0.6

The Wilcoxon signed rank test with continuity correction testing the difference in ranks between LDL after treatment and LDL before treatment suggested that the effect was negative, statistically significant, and very large (W = 443.50, p = 0.001, 95% confidence interval (CI) = -0.67, -0.23). It also showed that the difference in ranks between HDL after treatment and HDL before treatment suggested that the effect was negative, statistically not significant, and medium (W = 829.00, p = 0.053, 95% CI = -0.50, -3.73e-03). The test showed that the difference in ranks between total cholesterol after treatment and total cholesterol before treatment suggested that the effect was negative, statistically significant, and very large (W = 652.00, p < 0.001, 95% CI = -0.70,-0.33). The difference in ranks between TGs before the treatment and after the treatment suggested that the effect was positive, statistically not significant, and medium (W = 1724.50, p = 0.114, 95% CI = -0.05,0.44) (Tables 3, 4).

**Table 3:** Association between subclinical hypothyroidism and lipid profile of the participants. Median (IQR), Wilcoxon rank sum test. LDL = low-density lipoprotein; HDL = high-density lipoprotein; IQR = interquartile range

Characteristic	N	Overall, N = 208	Affected group, N = 99	Controlled group, N = 109	P-value
LDL before treatment	138	3.42 (2.61, 4.30)	4.00 (3.07, 4.86)	2.89 (2.45, 3.63)	<0.001
HDL before treatment	192	1.17 (0.99, 1.42)	1.12 (0.90, 1.37)	1.23 (1.05, 1.48)	0.012
Total cholesterol before treatment	202	4.87 (4.19, 5.55)	4.96 (4.20, 5.87)	4.72 (4.18, 5.35)	0.13
Triglyceride before treatment	202	1.06 (0.75, 1.58)	1.32 (0.85, 1.89)	0.94 (0.66, 1.27)	<0.001

**Table 4:** Difference in lipid profile in patients with subclinical hypothyroidism before and after treatment. P-values <0.05 are considered significant. LDL = low-density lipoprotein; HDL = high-density lipoprotein

Variable	Estimate	Number before treatment	Number after treatment	Statistic	P-value	Low 95% CI	High 95% CI
LDL	-0.400	68	74	444	0.001	-0.649	-0.159
HDL	0.049	71	87	1,449	0.053	-0.00001	0.095
Total cholesterol	0.484	77	93	2,198	<0.001	0.250	0.775
triglyceride	0.119	77	93	1,725	0.114	-0.029	0.269

**Figure 1:**
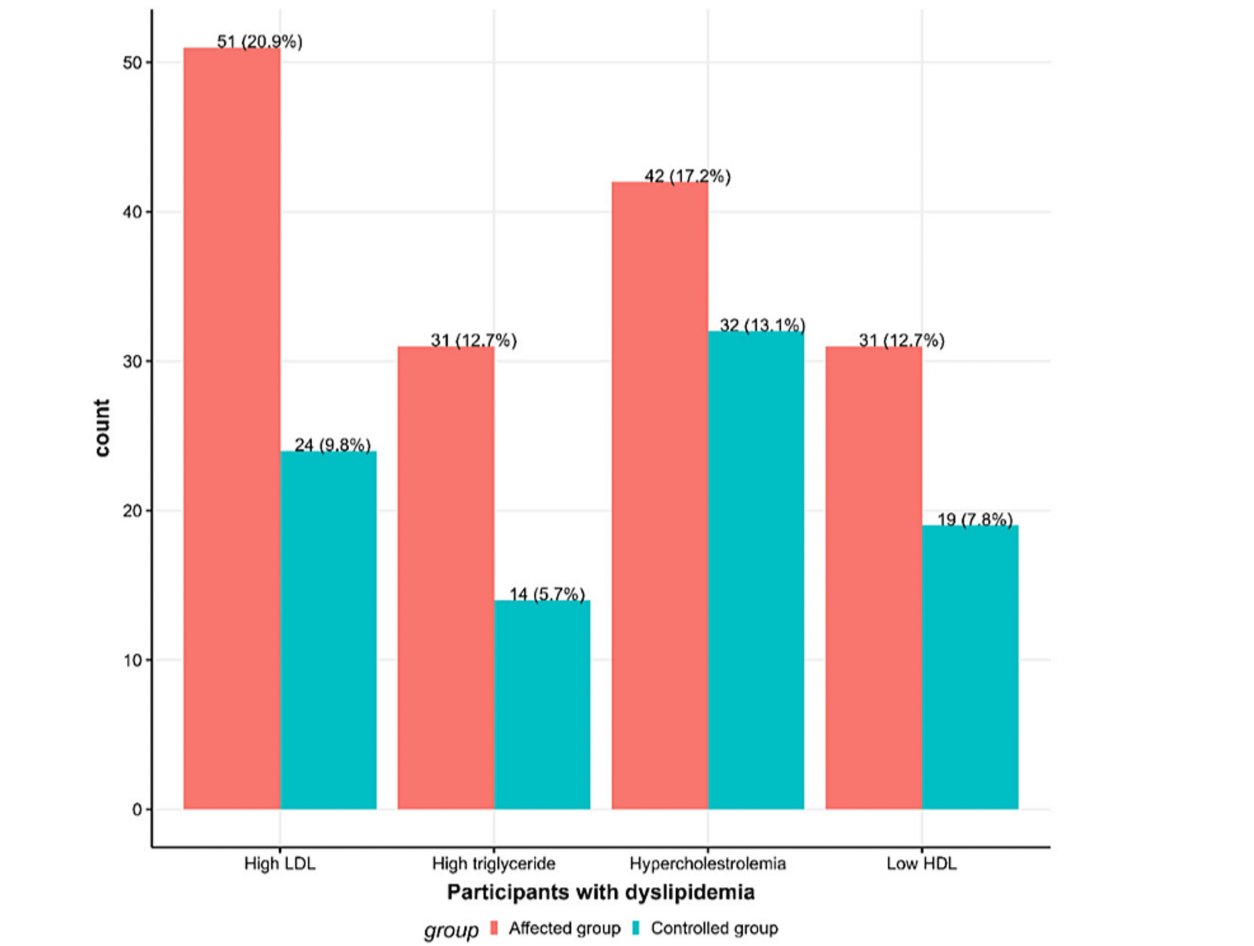
Distribution of dyslipidemia in the subclinical hypothyroidism and control groups. The affected group is shown in red, and the control group is shown in green. LDL = low-density lipoprotein; HDL = high-density lipoprotein

**Figure 2:**
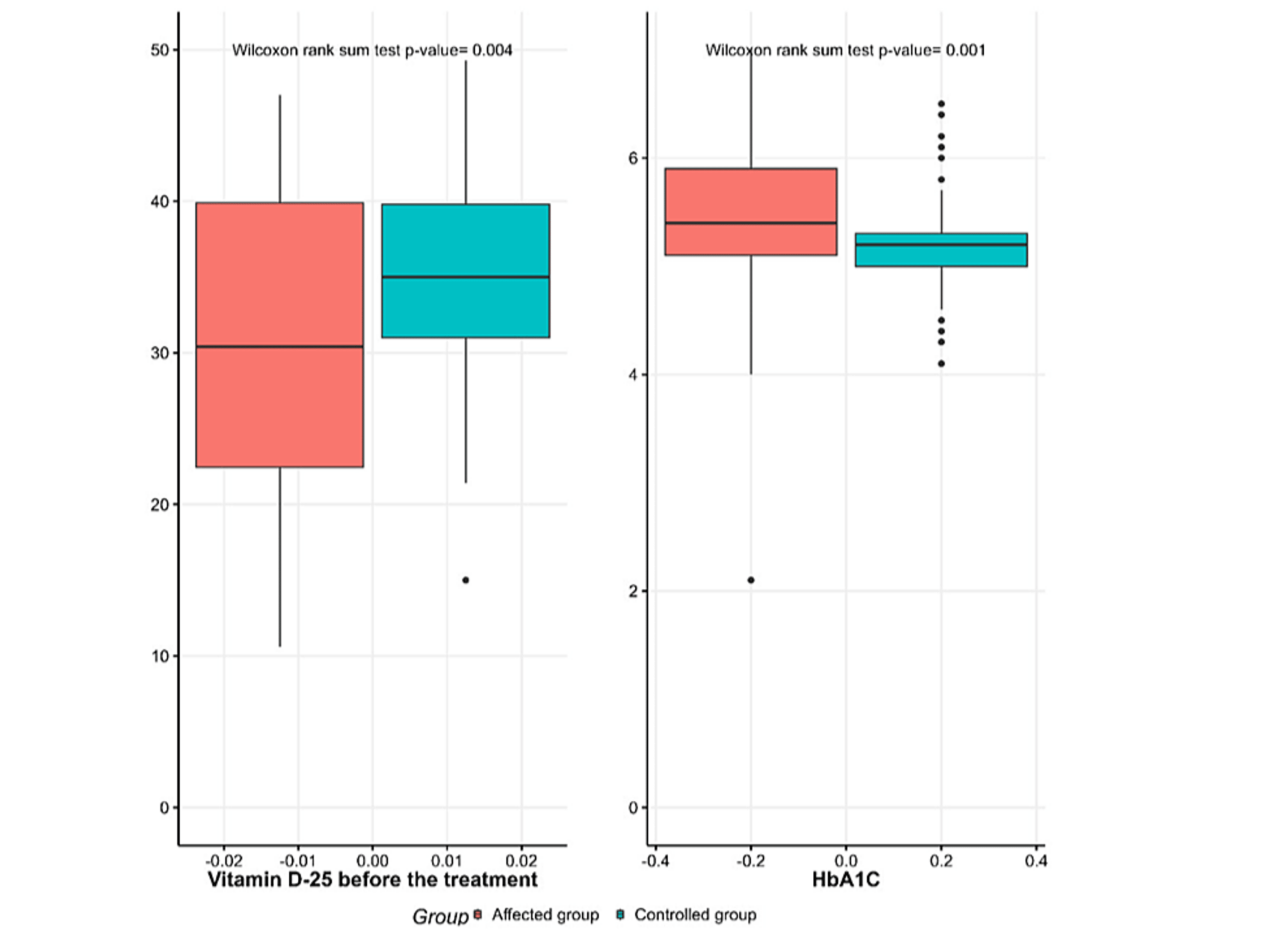
Median of vitamin D-25 and HbA1C levels in the subclinical hypothyroidism and control groups. The affected group is shown in red, and the control group is shown in green. HbA1C = glycated hemoglobin

Figure 1 illustrates dyslipidemia in the SCH and control group, with high percentages of high LDL, high TGs, low HDL, and hypercholesterolemia compared to the control group. Figure 2 shows significantly lower vitamin D-25 levels and higher HbA1C levels in the SCH group before treatment compared to the control group.

## Discussion

SCH is an asymptomatic condition that affects 10% of the population [11]. Despite being asymptomatic, it causes metabolic derangement [12,13] and increases all-cause mortality [14]. In this study, we found a significant association between SCH and the levels of vitamin D, HbA1c, LDL, and TG. Levothyroxine treatment significantly improves lipid profile with a main effect on LDL and cholesterol.

In this study, SCH was significantly associated with a decrease in vitamin D levels. The relative homology between vitamin D receptor and thyroid hormone receptor was noted in 1980 [15]. Later research showed an association between vitamin D deficiency and autoimmune thyroiditis [16]. Another study found that vitamin D supplementation delays the progression of autoimmune hypothyroidism, further emphasizing its role in the pathogenesis [17]. The findings are not confined to autoimmune hypothyroidism as the association was shown between SCH and vitamin D in children aged 6-24 months [18]. The mechanism causing this association is yet to be investigated.

Both fasting blood glucose and HbA1c were slightly higher among patients with SCH without reaching the diabetic or prediabetic range. The difference in HbA1c level between healthy subjects and patients with SCH was statistically significant. Hypothyroidism is more common among patients with type I and type II diabetes mellitus indicating a significant association [13,19]. It has been reported that thyroid dysfunction complicates 12.5% to 51.6% of diabetes cases with SCH being the most frequently reported dysfunction [20,21]. It is hypothesized that hypothyroidism causes insulin resistance and plays a role in the development of diabetes mellitus [22]. A recent study found a significant positive association between TSH and the homeostatic model assessment of insulin resistance [23]. On the other hand, a recent systematic review found no association between SCH and incident diabetes [24]. It should be noted that despite not reaching a diabetic level, HbA1c was significantly higher among patients with SCH in our sample. Whether this increase will later result in manifest diabetes mellitus is to be investigated.

There was no significant association between SCH and hypertension in our population. A recent meta-analysis showed that the association between SCH and hypertension is age dependent with significant association noted in the middle-age group but not in older females [25].

In this study, SCH was significantly associated with impaired lipid metabolism. Both LDL and TG were significantly higher among patients with SCH. While HDL was significantly lower in the same group. However, only LDL reached a pathological level. The association between hypothyroidism and dyslipidemia is widely studied in the literature [26-28]. A newly published meta-analysis showed a significant association between hypothyroidism and altered lipid profile in the adult population [29]. The decrease in the activity of lipoprotein lipase in adipose tissue and hepatic lipase is hypothesized to be the cause of elevated TG levels in SCH [30]. The high level of LDL may be attributed to decreased transcription of the LDL receptor gene [31]. Interestingly, it was suggested that dyslipidemia itself increases the risk of hypothyroidism [32]. On the other hand, the association between hypothyroidism and dyslipidemia is not universal and a lack of association is reported in the literature [33,34]. Dyslipidemia plays a role in increased cardiovascular disease in patients with SCH [35]. The benefit of treating SCH is debated in the literature and current guidelines suggest individualized decisions [14,36,37]. In our study, we used levothyroxine to treat patients with SCH. The treatment aimed to investigate its effect on lipid profile and indeed it improved LDL and cholesterol levels emphasizing the role of thyroid hormone in lipid metabolism [38].

This study has several limitations that should be considered. First, it was a retrospective case-control study, which means the data were collected from existing medical records rather than a prospective design. This can introduce biases and limitations in the data available. Second, the sample size, while reasonable, was still relatively small, especially for the SCH group. A larger sample size would provide more statistical power and confidence in the results. Third, the study only examined the association between SCH and lipid profile and did not evaluate the impact of levothyroxine treatment on lipid levels over time. Prospective studies following patients before and after treatment would provide more insight into the dynamic relationship. Finally, the study was conducted in a single healthcare system in Jeddah, Saudi Arabia, so the findings may not be fully generalizable to other populations or settings. Future research should aim to validate these results in larger, more diverse patient cohorts.

## Conclusions

Our study showed that SCH was significantly associated with lower levels of vitamin D and HDL. It was associated with higher levels of HbA1c, LDL, and TG levels. Only LDL and vitamin D fell within the pathological level. Levothyroxine treatment improved LDL and total cholesterol levels. Future studies should investigate the cost-effectiveness of treating SCH.
